# Prevalence and Species Distribution of *Candida* Clinical Isolates in a Tertiary Care Hospital in Ecuador Tested from January 2019 to February 2020

**DOI:** 10.3390/jof10050304

**Published:** 2024-04-24

**Authors:** Yessenia Acosta-Mosquera, Juan Carlos Tapia, Rubén Armas-González, María José Cáceres-Valdiviezo, Juan Carlos Fernández-Cadena, Derly Andrade-Molina

**Affiliations:** 1 Faculty of Medicine, Universidad Nacional del Nordeste, Corrientes 3400, Argentina; yessenia0510@yahoo.com; 2 Omics Science Laboratory, Faculty of Health Science, Universidad Espíritu Santo, Samborondon 092301, Ecuador; jtapian@unemi.edu.ec (J.C.T.); majocv97@gmail.com (M.J.C.-V.); 3 Instituto Interamericano de Cooperación para la Agricultura (IICA), Representación Ecuador-Proyecto-5CN-1RBT, Quito 170518, Ecuador; ruben.armas.consultor@iica.int; 4 Faculty of Health Science, Universidad Espíritu Santo, Samborondon 092301, Ecuador; 5 African Genome Center, University Mohammed VI Polytechnic (UM6P), Lot 660, Hay Moulay Rachid, Ben Guerir 43150, Morocco

**Keywords:** candidemia, *Candida*, epidemiology, Ecuador, NCA species, ITS phylogeny

## Abstract

The incidence of candidemia in healthcare centers is associated with high morbidity and mortality. Frequency varies significantly among regions, with some species being more prevalent than others in Latin America. In this study, 191 clinical *Candida* isolates were collected from a major hospital in Ecuador from January 2019 to February 2020 aiming to assess their prevalence and distribution. After data processing, 168 isolates characterized by the VITEK 2 system were subsequently identified by ITS sequencing. Results showed diverse *Candida* species distributions, with *C. albicans* and *C. tropicalis* being the most prevalent across different clinical sources. In hospitalized individuals, *C. tropicalis* (38%) and *C. albicans* (37%) were the most prevalent, followed by, *C. parapsilosis* (16%), *C. glabrata* (5%), and other non-*Candida albicans* (NCA) species (6%). Conversely, *C. parapsilosis* (48%), *C. albicans* (20%), and *C. glabrata* (14%), associated with candidemia, were the most common in blood and CSF. Additionally, uncommon NCA species such as *C. haemulonii*, *C. kefyr*, and *C. pelliculosa* were identified in Ecuador for the first time. Discrepancies in species identification were observed between the VITEK 2 system and ITS sequencing, coinciding at 85%. This highlights the need for ongoing surveillance and identification efforts in Ecuador’s clinical and epidemiological settings.

## 1. Introduction

The impact of fungal infections caused by *Candida* species on human health, besides being transcendental, is sometimes not widely recognized [1]. For instance, it is assumed that 90% of HIV-positive patients who are not being treated with highly active antiretroviral therapy (HAART) will develop either oropharyngeal or esophageal candidiasis [2]. Additionally, candidemia in hospitalized patients is the leading mycosis in tertiary care hospitals worldwide [3], mainly due to the emergence and prevalence of colonization of new non-*Candida albicans* (NCA) species. This emergence is driven by the increased use of prophylactic antifungals [4,5]. Considering the above, it becomes imperative to monitor infections caused by *Candida* spp. constantly. While this monitoring is routine in the USA, Europe, and Asia, epidemiological information on fungal infections caused by *Candida* spp. is scarce in Latin America, except Brazil [6,7].

A report suggests that the incidence of candidemia or invasive candidiasis in Latin America is mainly caused by *Candida albicans* (37.6%), followed by other NCA species such as *Candida parapsilosis* (26.5%), *Candida tropicalis* (17.6%) and *Nakaseomyces glabratus* (formerly referred to as *Candida glabrata*) (6.3%) [6]. Emerging infections by other NCA species such as *C. auris*, *C. dubliniensis*, *C. haemulonii*, *Kluyveromyces marxianus* (formerly referred to as *Candida kefyr*), *Pichia kudriavzevii* (formerly referred to as *Candida krusei*), *Clavispora lusitaniae* (formerly referred to as *Candida lusitaniae*), *Candida orthopsilosis*, *Wickerhamomyces anomalus* (formerly referred to as *Candida pelliculosa*) and *Diutina rugosa* (formerly referred to as *Candida rugosa*) have the potential to rapidly spread among critical patients and become dominant opportunistic pathogens in their populations [5]. Over the last two decades, the proportion of candidemia caused by NCA species has increased from 40% to 65%, largely related to improvements in diagnostic methods, such as molecular techniques incorporated into the routine diagnosis of fungemia [8]. Amplification of internal transcribed spacers (ITSs) is considered an effective method for confirming the identity and conducting phylogenetic analysis in *Candida* species [9].

The most common etiological agent of candidiasis has been *C. albicans*. However, recent studies have shown a shift in dominance from *C. albicans* to NCA species such as *C. tropicalis*, *C. parapsilosis*, *C. glabrata* (*Nakaseomyces glabratus*) and *C. krusei* (*Pichia kudriavzevii*) [10,11,12,13]. In 2003, a regional study with 2139 clinical isolated cases from Colombia, Ecuador, and Venezuela found that *C. albicans* was the most typical isolate (62%), followed by *C. parapsilosis* (11%), *C. tropicalis* (8.5%), and C. glabrata (*Nakaseomyces glabratus*) (3.5%) [14]. A decade later, a study on candidemia in Latin America estimated a rate of 0.90 cases per 1000 admissions in Ecuador, with 52.2% of cases due to *C. albicans*, 30.4% to *C. parapsilosis* and 10.9% to *C. tropicalis* [6]. However, the demographic distribution and the sample sizes were not representative of the countries. Another study in Ecuador employed ITS banding patterns and size to analyze 170 samples collected between January 2000 and August 2007 from the strain collection of the Clinical Mycology Laboratory of the Pontificia Universidad Católica del Ecuador (PUCE). The study identified *C. albicans* and *C. tropicalis* as the most prevalent strains [15]. Altogether, these studies reflect the changing landscape of candidiasis etiology, encouraging the need for local research to better understand its epidemiological behavior in Ecuador.

The aim of this study is to present the distribution and molecular characterization of *Candida* spp. from clinical isolates collected between January 2019 and February 2020 in a tertiary hospital in Guayaquil, Ecuador.

## 2. Materials and Methods

### 2.1. Clinical Samples and Yeast Isolation Culture

Clinical fungal isolates were obtained according to the standard microbiological protocols of the laboratory of microbiology at a tertiary hospital (Guayaquil, Ecuador) from January 2019 to February 2020. Fresh biological samples were examined to evaluate cell morphology and Gram-stain appearance. Yeast-like colonies were purified onto Sabouraud dextrose agar (SDA-Oxoid, ThermoScientific, Waltham, MA, USA) supplemented with chloramphenicol (0.05 g/L) and incubated at 37 °C for 24/48 h. Identification was achieved through subculture on a HardyCHROM™ Candida (CRITERION^®^, Hardy Diagnostics, Santa Maria, CA, USA), and samples were incubated at 37 °C for 48 h. To confirm *Candida* species, a YS card and the VITEK 2 system (bioMérieux, Inc., Hazelwood, MO, USA) were used following the manufacturer’s instructions. In brief, the colonies that had previously grown on Sabouraud medium at 35 °C for 24 h were suspended in a sterile physiological solution. The suspensions were prepared at 1.8–2.2 McFarland scale turbidity and measured using DensiChek (bioMérieux, Inc., Hazelwood, MO, USA), and the inoculum was dispensed on the YS card. The incubation time depended on the growth rate of organisms in the control well, whereas identification was made by comparison with the database.

### 2.2. DNA Extraction

Prior to DNA extraction, all fungi were cultured on SD agar at room temperature for 24/48 h. Each isolate was suspended in 400 μL of protoplast buffer (1.0 M sorbitol, 1% β-mercaptoethanol, 0.25 mg of lysozyme (Sigma-Aldrich, St. Louis, MO, USA) and 0.25 mg of lyticase (Sigma-Aldrich, St. Louis, MO, USA) and incubated at −80 °C overnight, followed by heat-shock incubation at 37 °C for 2 h. Fungal DNA was extracted using a PureLink^®^ Genomic DNA Mini Kit (Invitrogen, Waltham, MA, USA) following the manufacturer’s protocol and with subsequent modifications: the previously suspended samples in protoplast buffer were mixed with 180 μL digestion buffer and proteinase K. The samples were incubated for 30 min at 65 °C and RNAse A was added. Then, the mixtures were incubated at room temperature for 10 min. The samples were subjected to centrifugation at 10,000× *g* for 5 min and supernatants were collected into new Eppendorf tubes. Subsequently, they were mixed with 200 μL of 100% ethanol and vortexed for 5 s. Then the solutions were transferred to a spin column and centrifuged at 10,000× *g* for 1 min. This was followed by washing with 500 μL of wash buffer 1 and centrifugation at 10,000 rpm for 1 min. Then, 500 μL of wash buffer 2 was added to the spin columns and centrifuged at 10,000 rpm for 3 min. The DNA was eluted by adding 100 μL of ultrapure nuclease-free water incubated at room temperature for 3 min and centrifuged at 13,000 rpm for 1 min. The genetic material was then stored at −20 °C until further use. Purity of the DNA was evaluated by spectrophotometric A_260/280_ ratio, while the concentration was calculated according to A_260_. DNA integrity was assessed by 1% agarose gel electrophoresis in 1X TAE buffer (Tris, Acetate, EDTA) supplemented with SYBR green nucleic acid stain (Invitrogen) by comparing the intensity and molecular weight band of DNA with a 100 bp ladder (Trackit-Invitrogen). Electrophoresis conditions were performed at 100 volts, 35 milliamps.

### 2.3. Molecular Characterization of Candida Clinical Isolates by ITS Regions

After DNA extraction, the isolates were identified by the amplification of the internal transcribed spacers of rDNA using ITS-1 (5′-TCC GTA GGT GAA CCT GCG G-3′) and ITS-4 universal primers (5′-TCC TCC GCT TAT TGA TAT GC-3′) [16]. PCR conditions were performed in a final volume of 30 μL with a final concentration of 0.25 mM dNTPs, 2.5 mM MgCl_2_, 2 μM primers, 2U Taq polymerase and 1X PCR buffer. The program included initial denaturation at 95 °C for 5 min, followed by 35 cycles with denaturation at 94 °C for 60 s, annealing at 51 °C for 80 s and extension at 72 °C for 60 s, with a final extension at 72 °C for 10 min. Further sample processing resulted in highly diverse representative strains. Purified amplicons were sequenced in Macrogen (Seoul, Republic of Korea) and edited in Geneious Prime 2023 software (version 2023.0.4) to obtain a consensus sequence. Taxonomic affiliation was confirmed by the BLAST alignment algorithm (https://blast.ncbi.nlm.nih.gov/Blast.cgi, accessed on 22 September 2023). ITS sequences were deposited in Bioproject PRJNA1077920 (Genbank ID: PP503965-PP504130). Figures were constructed in R version 4.2.2 for Mac using the ggplot2 package Version 3.5.0 [17].

## 3. Results

### 3.1. Origin of Candida Strains

**Figure 1:**
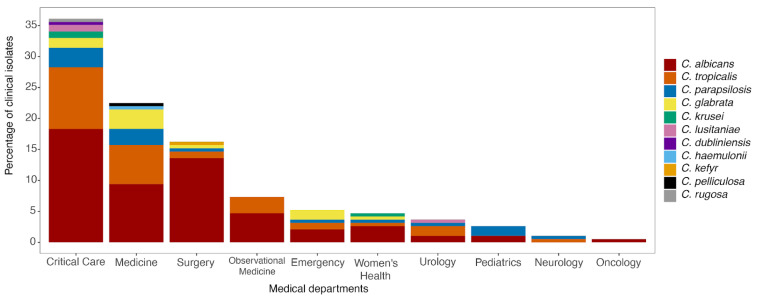
Total distribution of 191 *Candida* species isolated across different hospital departments from a tertiary hospital in Guayaquil, Ecuador between January 2019 and February 2020.

From January 2019 to February 2020, a total of 191 *Candida* isolates were identified from patients admitted to different departments of the tertiary care hospital in Ecuador. *C. albicans* was isolated from the majority of hospital departments (53%), with the highest incidence reported in the critical care department (18%, *n* = 35), followed by the surgery department (14%, *n* = 26), general medicine department (10%, *n* = 18), observational medicine department (5%, *n* = 9), women’s health department (3%, *n* = 5), and the emergency department (2%, *n* = 4). The remaining 3% was found among the departments of urology, pediatrics and oncology. With respect to NCA species (47%), *C. tropicalis* and *C. parapsilopsis* were identified as the predominant species, accounting for 52% and 22% of the total NCA, respectively. They were mainly found in critical care, surgery and observational medicine departments. The third-commonest NCA was *C. glabrata* (14%), identified in the departments of general medicine (43%), emergency (21%) and critical care (21%). *C. lusitanae* and *C. krusei* represented 4% of all isolates, while the remaining five included uncommon NCA species (*C. pelliculosa*, *C. haemulonii*, *C. kefyr*, *C. dubliniensis* and *C. rugosa*) (Figure 1).

### 3.2. Molecular Characterization of Candida Strains and Source Distribution

**Figure 2:**
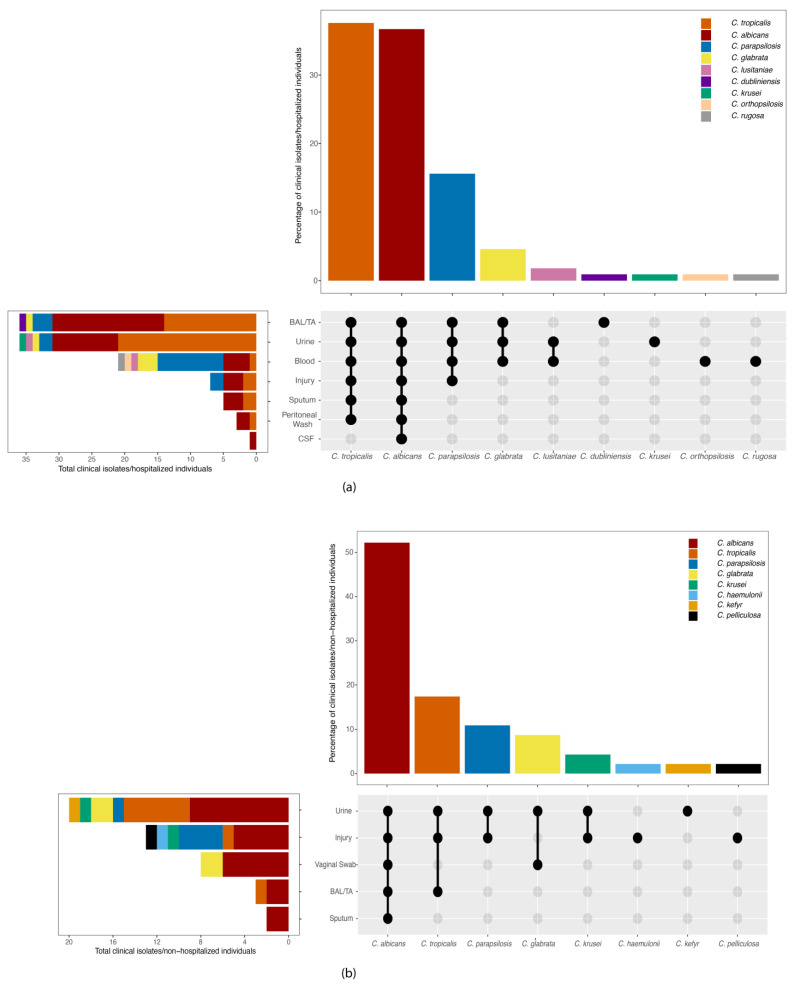
UpSet plot: distribution of *Candida* species by clinical samples from (**a**) hospitalized and (**b**) non-hospitalized individuals collected from a tertiary hospital in Guayaquil, Ecuador between January 2019 and February 2020. BAL/TA: bronchoalveolar lavage/tracheal aspirate, CSF: cerebrospinal fluid.

Following sequencing data processing, 168 *Candida* spp. that originated from diverse clinical sources among hospitalized and non-hospitalized individuals were confirmed. Cases of candidemia were detected in hospitalized individuals, particularly isolates from blood and cerebrospinal fluid (31%), while the remaining 69% were recognized as colonization cases, distributed as follows: 33% (*n* = 36) from bronchoalveolar lavage/tracheal aspirate (BAL/TA), 33% (*n* = 36) from urine, 6% (*n* = 7) from injury, 5% (*n* = 5) from sputum, and 3% (*n* = 3) from peritoneal wash (Figure 2a). In contrast, isolates from non-hospitalized individuals showed that 43% originated from urine (*n* = 20), 28% from injury (*n* = 13), 17% from vaginal swabs (*n* = 8), 7% from BAL/TA (*n* = 3), and 4% from sputum (*n* = 2) (Figure 2b).

The molecular characterization of *Candida* strains involved the sequencing of the ITS region in all clinical isolates. The results showed a diverse distribution of *Candida* species among both hospitalized and non-hospitalized individuals. For hospitalized individuals, the predominant *Candida* species were *C. albicans* (37%) and *C. tropicalis* (38%), followed by *C. parapsilosis* (16%), *C. glabrata* (5%), *C. lusitaniae* (2%), *C. dubliniensis* (2%). The remaining 4% comprised *C. krusei*, *C. rugosa* and *C. orthopsilosis.* Among the different specimen sources, the greatest abundance of isolates was detected in BAL/TA and urine samples. For BAL/TA (*n* = 36), *C. albicans* was the most prevalent (*n* = 17), followed by *C. tropicalis* (*n* = 14), *C. parapsilosis* (*n* = 3), *C. glabrata* (*n* = 1) and *C. dubliniensis* (*n* = 1). For urine specimens (*n* = 36), *C. tropicalis* was the most common species (*n* = 21), followed by *C. albicans* (*n* = 10), and *C. parapsilosis* (*n* = 2), with C. *glabrata*, *C. lusitaniae*, *C. krusei* each having one isolate. The other most representative source was blood (*n* = 20), from which 48% of isolates were *C. parapsilosis* (*n* = 10), followed by 19% *C. albicans* (*n* = 4), 14% *C. glabrata* (*n* = 3), and the remaining 20% equally distributed among *C. lusitaniae*, *C. rugosa* and *C. orthopsilosis.* Additionally, *C. albicans* was identified in a CSF source linked to candidemia. In injuries (*n* = 7), *C. albicans* (*n* = 3), *C. parapsilosis* (*n* = 2) and *C. tropicalis* (*n* = 2) were identified, while peritoneal wash and sputum sources showed *C. albicans* and *C. tropicalis* with three and five isolates, respectively (Figure 2a).

For non-hospitalized individuals, *C. albicans* was the most common species (52%), followed by *C. tropicalis* (17%), *C. parapsilosis* (11%), *C. glabrata* (9%), and *C. krusei* (4%), and the remaining 6% were rare NCA species, such as *C. haemulonii*, *C. kefyr*, and *C. pelliculosa.* Among the sources that were analyzed, urine (*n* = 20) emerged as the most frequent source of isolates. Within these urine samples, *C. albicans* was detected in nine cases, followed by *C. tropicalis* (*n* = 6), *C. glabrata* (*n* = 2) and one each of *C. parapsilosis*, *C. krusei*, and *C. kefyr*. In injury cases (*n* = 13), *C. albicans* and *C. parapsilosis* were predominant (*n* = 5, *n* = 4 respectively)*,* with singular occurrences of *C. tropicalis* and *C. krusei* and two species identified as rare NCA, namely, *C. haemulonii* and *C. pelliculosa*. BAL/TA yielded isolates of both *C. albicans* and *C. tropicalis*. Regarding vaginal swabs, only *C. albicans* (*n* = 6) and *C. glabrata* (*n* = 2) were identified, all from non-hospitalized individuals. Finally, two sputum isolates were both identified as *C. albicans* (Figure 2b).

Notably, from the environmental catheter source, *C. parapsilosis* (*n* = 5) and *C. albicans* (*n* = 3) were identified. Among isolates with unreported sources (*n* = 5), *C. tropicalis* and *C. orthopsilosis* were detected in two and three cases, respectively.

### 3.3. Comparison of VITEK 2 System and ITS Sequencing

Although most of the results from VITEK 2 (bioMérieux) and ITS sequencing agreed, there were differences, particularly in identifying *C. orthopsilosis*. Initially, four isolates that were first recognized as *C. parapsilosis* by VITEK 2 were identified as *C. orthopsilosis* using ITS sequencing. Kappa coefficient (k), standard error and 95% confidence intervals were calculated to assess the level of agreement between phenotypic and molecular methods. Results indicated that both methods had an almost perfect level of agreement (0.859), and there seemed to be no significant standard error between them (0.033; 0.785–0.916).

## 4. Discussion

Candidiasis and candidemia are major healthcare-associated fungal infectious diseases. The epidemiology and prevalence of *Candida* infectious diseases are influenced by factors such as hospital treatment approaches, patient-specific risk factors, antifungal resistance, and geographic location [6,18,19,20,21]. This is the first report on the epidemiological situation in Ecuador to analyze the distribution of *Candida* clinical isolates for a year, whereby the species were characterized through ITS sequencing. We identified a wide variety of species, including the most abundant species: *C. albicans*, *C. tropicalis*, *C. parapsilosis* and *C. glabrata*. However, other emerging and rare NCA species were also detected, of which some are reported for the first time in the country and even in Latin America.

In our study, *C. albicans* was the most prevalent species (53% of total isolates). These results align with the species distribution observed in healthcare centers across different countries, including Shanghai, China; Beirut, Lebanon; Apulia, Italy; and southern Brazil [22,23,24,25]. In these studies, *C. albicans* was the most frequently isolated species within medical departments, followed by *C. tropicalis* and *C. parapsilosis*. The results indicate that *C. albicans* is the foremost *Candida* species. Nevertheless, there is a potential for NCA to surpass it in prevalence. It is important to approach these findings with caution. The observed prevalence rates of *Candida* species may be influenced by the frequency and scope of the conducted microbiological analyses across different departments, as well as the biological sources.

Diverse regional variations in the prevalence of *Candida* species have been extensively discussed in the literature, in which *C. albicans* (18–76%), *C. tropicalis* (10–39%), *C. parapsilosis* (5–49%) and *C. glabrata* (15–25%) are predominant [26,27,28,29,30,31,32,33,34,35,36]. Our study showed a high shared predominance of isolates from hospitalized and non-hospitalized individuals between *C. albicans* (40% vs. 52%) and *C. tropicalis* (35% vs. 17%), followed by *C. parapsilosis* (14% vs. 11%), *C. glabrata* (5% vs. 9%) and emerging NCA species (7% vs. 10%). By contrast, a decade ago, distinctive distribution in Ecuador was indicated, with 52.2% of invasive candidiasis attributed to *C. albicans*, *C. parapsilosis* (30.4%), *C. tropicalis* (10.9%), *C. glabrata* (4.3%), and *C. guilliermondii* (2.2%) [6]. Discrepancies with our study were not only in the distribution and prevalence of *Candida* species but also in the identification of new *Candida* species.

Recent decades have witnessed a decline in *C. albicans* prevalence, dropping to as low as 44% in some countries [27,36,37,38,39]. Emerging NCA species have increasingly taken precedence, especially in intensive care unit (ICU) hospitalizations, and both hospital and community-acquired infections have become more prevalent [40]. In our study, *C. tropicalis* emerged as a predominant NCA identified mainly in urine and BAL/TA samples, whereas *C. parapsilosis* was the second-commonest NCA species identified, particularly isolated from blood. Similar prevalence of both *C. tropicalis* and *C. parapsilosis* was observed in a longitudinal study conducted in Colombia in tertiary health institutions, in which these species were also identified in respiratory samples and blood [41].

The prevalence patterns of *C. glabrata* and *C. krusei* are of special importance in public health due to their increasing reported resistance to commonly used antifungal agents [42,43,44,45,46] and their rapid dissemination, particularly in immunocompromised patients [27,47,48]. Our study found a prevalence of 5% of *C. glabrata* in hospitalized individuals, of which three isolates originated from blood samples. Among non-hospitalized individuals, *C. glabrata* was identified in urine and vaginal swabs, with a prevalence of 9%. These findings differ from the annual proportion of *C. glabrata* bloodstream isolates among all *Candida* reported by Won et al., who observed an increase in infection caused by *C. glabrata* from 11.7% to 23.9% (mean 18.6%) [49]. Another study reported *C. glabrata* as the most relevant etiologic agent among NCA species in vulvovaginal and urinary tract infections [50,51,52]. Concerning *C. krusei*, although it was not detected in blood isolates, we identified four isolates that originated from urine and wound samples. These findings align with the epidemiology of candidemia in Latin America, which show a prevalence of 2.7 to 3.3% [6,53]. This contrasts with reports from Asian and African regions where the prevalence of *C. krusei* increased from 10% to 33%, particularly in bloodstream isolates [48,54,55]. These findings underscore the critical public health significance of monitoring prevalence and treatment strategies, especially regarding *Candida* species with increasing resistance and rapid spread in clinical settings.

Conventional diagnostic methods pose significant challenges to the accurate identification of *Candida* species, primarily due to their low sensitivity and prolonged processing time. For instance, *C. auris* is often misidentified with closely related species such as *C. haemulonii* when employing these methods [56]. To address these limitations, targeted genome sequencing has emerged as a promising approach for the development of rapid and accurate diagnostic assays [57]. In this study, the ITS region sequencing allowed the identification of emerging NCA species, including *C. dubliniensis*, *C. haemulonii*, *C. kefyr*, *C. krusei*, *C. lusitaniae*, *C. orthopsilosis*, *C. pelliculosa*, and *C. rugosa*. Moreover, the identity of *C. haemulonii* was confirmed through whole-genome sequencing. Notably, we found no evidence of *Candida auris* presence in this hospital, contrasting with reports in other Latin American countries. Thus far, *C. auris* has not been documented in hospital environments or the resistance antimicrobial monitoring (RAM) system overseen by the Ecuadorian government.

Our findings not only contribute to expanding our knowledge of the fungal diversity of Ecuador but also highlight the importance of implementing molecular epidemiology schemes. These schemes would enable us to track rare NCA species occurrence and to study other important factors such as multidrug resistance (MDR) and virulence gene profiles associated with possible outbreaks [58,59,60,61]. Given the scarcity and insufficiency of epidemiological data on circulating *Candida* species within the country, it becomes imperative to conduct regional characterization to delineate typical distribution patterns and resistance rates. This is essential for establishing appropriate measures for infection control, managing outbreaks, implementing routine diagnostics, and conducting surveillance of patients at risk of invasive candidiasis.

Although the statistical agreement between VITEK 2 and ITS sequencing methods reached 85%, it is crucial to recognize the potential consequences of misidentification, as highlighted in some studies [62,63,64]. Misclassification of isolates raises significant concern, given the potential association with increased antimicrobial resistance and even more so when the gold standard diagnostic method in Ecuador is the VITEK 2 system test. Ensuring accurate identification of pathological agents is imperative to facilitate the implementation of appropriate control measures and treatments prescribed.

The global distribution of *Candida* spp. plays a significant role in the epidemiology of these fungal infections, commonly associated with a notable prevalence of *C. albicans*, *C. glabrata*, *C. tropicalis*, *C. parapsilosis*, and *C. krusei* [36,40]. Our research findings are consistent with this global distribution, as observed in the distribution trends of these species around the time of data collection, highlighting the dominance of *C. albicans*, *C. tropicalis* and *C. parapsilosis* and the emergence of non-typical *Candida* species throughout the year.

Recently, the World Health Organization Fungal Priority Pathogens List (WHO FPPL) categorized *C. albicans* as a critical priority group and included *C. tropicalis*, *C. parapsilosis* and *C. glabrata* in the high-priority group of fungal pathogens [65]. This classification aims to generate standard guidelines focused on specific preventive measures to strengthen laboratory capacities, surveillance, sustainable investments in research, development, innovation, and public health interventions. There is a growing need to adopt molecular techniques as part of diagnostic new strategies and genomic surveillance, such as real-time PCR diagnostics and next-generation sequencing (NGS) identification-based clinical tests for fungal species to decrease mortality and reduce misuse of antifungals [66,67,68,69,70]. However, molecular approach implementation such as fungal whole-genome-sequencing (WGS) in clinical microbiology is a real challenge in Ecuador. The primary areas of global response to fungal infections and antifungal resistance in the country are not considered within public policies.

Our study has several limitations. Firstly, there is a lack of information regarding clinical history underlying patients’ medical conditions and comprehensive healthcare records detailing treatments received. Secondly, the antifungal susceptibility profile of *Candida* isolates was incomplete, hindering a thorough analysis of the virulence characteristics of these clinical isolates. This information is crucial for implementing effective strategies to control outbreaks of resistant *Candida* species.

## 5. Conclusions

*C. albicans* was the most frequently isolated species, particularly predominant in critical care and hospitalized individuals between January 2019 and February 2020 in a tertiary hospital of Ecuador. NCA species made up 46% of the isolates, with a high prevalence of *C. tropicalis*, *C. parapsilosis* and *C. glabrata*. During this research period, species considered rare NCA were reported (*C. dubliniensis*, *C. krusei*, *C. lusitaniae*, and *C. rugosa*). In addition, the presence of *C. haemulonii*, *C. kefyr* and *C. pelliculosa* species are reported for the first time in the country. We recognize the inherent limitations of the research, such as the absence of comprehensive clinical, epidemiological, and antifungal susceptibility data. These highlight the immediate necessity of implementing public health policies focused on priority pathogenic fungi. These challenges are particular to Ecuador, where there is a notable shortfall in infrastructure, equipment, and an established fungal surveillance program. Despite these limitations, this study provides valuable insight into the current state of *Candida* species within the healthcare system of Ecuador’s most populous city.

## Data Availability

The original contributions presented in the study are included in the article, further inquiries can be directed to the corresponding author.
